# Supplementary material for: MetaRibo-Seq measures translation in microbiomes
Source: Nat Commun. 2020 Jun 29;11:3268. doi: 10.1038/s41467-020-17081-z (PMC7324362; doi:10.1038/s41467-020-17081-z)
Supplement: Supplementary file 10 — Supplementary Data 7 [file 41467_2020_17081_MOESM10_ESM.zip › File2/Confidence_High_Taxonomy/31331_out.krona.html]

Javascript must be enabled to view this page.

members
magnitude
magnitudeUnassigned
count
unassigned
taxon
rank

31331\_out

5

5
2
superkingdom

5
1239
phylum

class
5
186801

order
186802
5

186803
5
family

genus
5
841

species

SRS012969\_contig\_number\_4507
1
2293144

species
4

SRS024132\_contig\_number\_10342SRS051031\_contig\_number\_25404SRS055017\_contig\_number\_15003SRS143722\_contig\_number\_1274
1897017
